# Comparing Xe^+^pFIB and Ga^+^FIB for TEM sample preparation of Al alloys: Minimising FIB‐induced artefacts

**DOI:** 10.1111/jmi.12983

**Published:** 2020-12-24

**Authors:** Xiangli Zhong, C. Austin Wade, Philip J. Withers, Xiaorong Zhou, Changrun Cai, Sarah J. Haigh, M. Grace Burke

**Affiliations:** ^1^ Department of Materials University of Manchester Manchester UK; ^2^ Department of Materials, Materials Performance Centre University of Manchester Manchester UK; ^3^ Department of Materials, Henry Royce Institute University of Manchester Manchester UK

**Keywords:** aluminium, artefacts, damage, FIB, pFIB, STEM, TEM

## Abstract

Recently, the dual beam Xe^+^ plasma focused ion beam (Xe^+^pFIB) instrument has attracted increasing interest for site‐specific transmission electron microscopy (TEM) sample preparation for a local region of interest as it shows several potential benefits compared to conventional Ga^+^FIB milling. Nevertheless, challenges and questions remain especially in terms of FIB‐induced artefacts, which hinder reliable S/TEM microstructural and compositional analysis. Here we examine the efficacy of using Xe^+^ pFIB as compared with conventional Ga^+^ FIB for TEM sample preparation of Al alloys. Three potential source of specimen preparation artefacts were examined, namely: (1) implantation‐induced defects such as amophisation, dislocations, or ‘bubble’ formation in the near‐surface region resulting from ion bombardment of the sample by the incident beam; (2) compositional artefacts due to implantation of the source ions and (3) material redeposition due to the milling process. It is shown that Xe^+^pFIB milling is able to produce improved STEM/TEM samples compared to those produced by Ga^+^ milling, and is therefore the preferred specimen preparation route. Strategies for minimising the artefacts induced by Xe^+^pFIB and Ga^+^FIB are also proposed.

**Lay Description:**

*FIB (focused ion beam) instruments have become one of the most important systems in the preparation of site‐specific TEM specimens, which are typically 50‐100 nm in thickness. TEM specimen preparation of Al alloys is particularly challenging, as convention Ga‐ion FIB produces artefacts in these materials that make microstructural analysis difficult or impossible. Recently, the use of noble gas ion sources, such as Xe, has markedly improved milling speeds and is being used for the preparation of various materials. Hence, it is necessary to investigate the structural defects formed during FIB milling and assess the ion‐induced chemical contamination in these TEM samples. Here we explore the feasibility and efficiency of using Xe^+^PFIB as a TEM sample preparation route for Al alloys in comparison with the conventional Ga+FIB*.

## 1 INTRODUCTION

The preparation of electron‐transparent transmission electron microscopy (TEM) samples from a site‐specific region of interest in a material using focused ion beam (FIB) milling has become one of the most important sample preparation routes. A high‐quality sample is critical for reliable TEM analysis for a very wide range of functional and structural materials.^1^ However, artefacts induced during the FIB milling process can hinder analysis and may yield misleading results.

Conventional Ga^+^ FIB processing is known to produce defects caused by the interaction of energetic Ga^+^ ions with the sample,^2^, ^3^, ^4^ for example, amorphisation of Si and diamond during Ga^+^FIB milling,^5^, ^6^, ^7^ phase changes observed in austenitic stainless steels,^8^ hydrides in Zr TEM samples,^9^ and Cu_3_Ga intermetallic phase (under normal incidence) in nanograin Cu samples.^3^, ^10^ During Ga^+^FIB milling of Al, implanted Ga tends to decorate the grain boundaries (GBs) and this may induce misleading or incorrect results for segregation studies.^11^ This is perhaps not surprising given that liquid Ga on an Al surface rapidly penetrates along the GBs, resulting in a very rapid, dramatic loss of Al ductility^12^ via liquid metal embrittlement (LME). Unocic et al examined the effect of Ar ion polishing as a final cleaning strategy but concluded that the optimum solution may be to avoid the use of Ga ion beams entirely.^11^ Apart from Ga ion‐induced chemical artefacts, other major concerns for conventional Ga^+^FIB milling of Al alloys are ion beam‐induced structural changes (amorphisation and dislocation loops etc),^8^, ^13^ as well as material redeposition.^14^ In spite of these issues, the conventional Ga^+^FIB has been the preferred method of site‐specific TEM sample preparation^15^ due to lack of other suitable local methods. Given these difficulties, the investigation of alternative methods for site‐specific TEM sample preparation in Al alloys is worthwhile.

It has been demonstrated that Xe+pFIB instruments can be successfully used for preparation of electron‐transparent TEM specimens of various materials.^16^, ^17^ However, the use of Xe pFIB for Al alloys has not been explored. Whilst the conventional Ga+ FIBs are still widely employed for TEM sample preparation, there is a need to understand the benefits and limitation for pFIB‐prepared TEM samples. Therefore, we have examined the advantages and disadvantages of Xe pFIB for Al alloys relative to the established Ga+FIB TEM sample preparation method. pFIBDual beam Xe^+^ plasma FIB‐SEM systems are well‐known for their large area milling capabilities compared to Ga^+^FIB.^17^, ^18^, ^19^, ^20^ The inductively coupled Xenon plasma ion source (ICP) in the Xe^+^pFIB column yields a higher ion currents (microamps compared to nanoamps for Ga^+^FIB) whereas the heavier Xe^+^ ions also contribute to a higher sputtering rate compared to Ga^+^ ions.^20^, ^21^, ^22^ Xe^+^pFIB instruments have smaller probe sizes than Ga^+^ beams at high ion currents, but larger probe sizes at currents less than 20 nA.^23^ Since Xe is a noble gas, it is unlikely to form a chemical bond with the Al, which can be an advantage over conventional Ga^+^FIB. Relatively few studies have reported the feasibility and limitations of using Xe^+^pFIB for TEM sample preparation. MacLaren et al demonstrated the feasibility of the Xe^+^pFIB for TEM sample preparation of oxide thin films and achieved atomic resolution STEM imaging.^16^, ^17^ Giannuzzi and Smith^24^ reported that Xe^+^pFIB milling produced a thinner amorphous damage layer on Si than conventional Ga^+^FIB. Xiao et al^25^ studied the effect of Ga^+^ milling and Xe^+^ final milling on mechanical responses of 7 μm (dia.) pillars and found that Xe^+^‐prepared pillars had higher yield strengths than Ga^+^‐prepared pillars because it avoided Ga segregation on the grain boundary that reduced the strength of polycrystalline aluminium pillars. However, questions remain as to the suitability of Xe^+^pFIB for the preparation of Al alloy TEM specimens, such as compositional contamination, structural changes (eg new phases),^26^, ^27^ ion‐induced damage (eg dislocation loops),^28^ and amorphisation,^2^, ^29^, ^30^ Xe ‘bubble’ or precipitate formation,^27^, ^31^, ^32^ as well as levels of redeposition.^5^ ^,^ ^14^

The aim of this paper is to explore the feasibility, advantages and limitations of Al alloy TEM sample preparation using Xe^+^pFIB compared with conventional Ga^+^FIB milling using similar milling parameters. In the first part of this paper, a model polycrystalline ‘pure’ Al specimen was used to revisit the nature of Ga^+^FIB‐induced artefacts and those generated during Xe^+^pFIB milling for the same sample. Procedures for optimising TEM specimen preparation to minimise the artefacts are proposed. We then apply the methodology to a ‘real’ commercial Al‐Zn‐Mg alloy (AA7108‐T6) to compare TEM samples produced by Xe^+^pFIB to previous results obtained for a sample prepared using conventional Ga^+^FIB. Finally, we examine the potential of the Xe^+^pFIB for site‐specific large area TEM sample preparation, which is known to be impractical using conventional Ga^+^FIB.

## 2 EXPERIMENTAL METHODS

### 2.1 Materials

Cold‐rolled and annealed commercially pure (99.94% Al with 10 ppm Mg, 20 ppm Fe, 50 ppm Cu and 480 ppm Si; Krupp VDM GmbH) polycrystalline Al sheet (grain size of 2‐3 μm) was used as a model sample.

**FIGURE 1:**
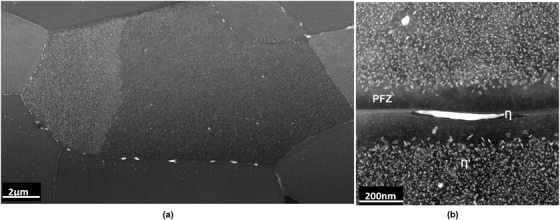
HAADF STEM images of the AA7108‐T6 microstructure: (a) Mg‐Zn (η’) distributed throughout the matrix and intergranular MgZn_2_ (η), (b) higher magnification of (a) showing intergranular MgZn_2_ (η) with an η’ precipitate‐free zone surrounding the grain boundary, and fine intragranular η’ precipitates

By way of an exemplar ‘real‐life’ sample, a commercial grade Al alloy AA7108‐T6 was chosen as an exemplar `real‐life' sample which have the composition (wt.%) of: Al ‐ 0.1Si ‐ 0.15Fe ‐ 0.05Cu ‐ 0.04Mn ‐ 0.75Mg ‐ 4.85Zn ‐ 0.03Cr ‐ 0.03Ti ‐ 0.005Pb ‐ 0.17Zr. This alloy had been aged (T6 condition) to produce a microstructure that consisted of fine Mg‐Zn η’ precipitates distributed throughout the matrix and coarser η‐MgZn_2_ intergranular precipitates (Figure 1). The bulk sample was mechanically cut and then metallographically polished. The AA7108‐T6 alloy had been exposed in a 3.5% NaCl‐H_2_O solution for 7 hours as part of a corrosion test. This specimen was selected to assess the preparation of large‐area TEM specimens with complex metal/oxide interfaces.

### 2.2 TEM preparation protocol

Two types of FIB specimens were prepared: (1) conventional ‘lift‐out’ type specimens and (2) ‘needle’ or pin‐type specimens similar to those used for atom probe tomography or high‐resolution X‐ray tomography.

All specimens were generated using common procedures for conventional FIB TEM preparation^33^, ^34^ in which a layer of protective Pt from a gas injection system (GIS) is deposited on the sample surface over the region of interest, followed by rough trench milling and then lifting out the lamella for attachment to a copper grid. Final thinning was then performed to achieve electron transparency for subsequent TEM analyses. The milling parameters were kept similar for both Ga^+^FIB and Xe^+^pFIB, and are listed in Table 1. Pin‐type samples were also prepared using Xe^+^pFIB‐SEM (ThermoFisher Helios Plasma FIB) and a conventional Ga^+^FIB‐SEM (ThermoFisher Helios FIB) systems.

**TABLE 1:** Ga+FIB and Xe+pFIB parameters for Al TEM sample preparation

TEM sample preparation system	Rough milling/trenching (nA/kV)	Progressive thinning (nA/kV)	Final thinning (nA/kV)	Final cleaning (pA/kV)
Ga^+^FIB	9.2‐20/30	2.5‐0.23/30	0.23/30	27/5
Xe^+^pFIB	15‐180/30	6.7‐1.8/30	0.23/30	27/5

### 2.3 FIB, TEM/STEM and STEM‐EDX analyses

The extent of ion‐induced artefacts in the electron‐transparent FIB‐prepared TEM specimens was characterised by TEM imaging and STEM‐EDX microanalysis. A Thermo Fisher Scientific Talos F200X FEG analytical S/TEM equipped with 4 silicon drift detectors (SDD) for energy dispersive X‐ray (EDX) spectroscopy operated at 200 kV was used for Xe and Ga elemental analysis. The scanning transmission electron microscopy (STEM) annular dark‐field imaging was performed at camera lengths ranging from 160 to 260 mm with ADF collection angles ranging from 67 to 200 mrad, encompassing medium‐angle and high‐angle annular dark‐field (MAADF and HAADF, respectively) modes. STEM‐EDX spectrum image (SI) datasets were collected with dwell time of 6.25 μs per pixel [livetime: 1.2 × 10^3^ seconds (20 minutes)] and a pixel size ranging from 0.2 to 5 nm. Data analysis was performed using the Thermo Fisher Scientific Velox (V2.8) software. Elemental maps were extracted from the background‐subtracted and deconvoluted SI datasets. Quantification of the SI was performed using Cliff Lorimer analysis. Conventional TEM images were also acquired using a Phillips CM20 TEM operated at 200 kV to assess the extent of ion‐induced damage.

## 3 RESULTS AND DISCUSSIONS

### 3.1 Commercially pure Al ‘model’ alloy

TEM characterisation of the FIB‐prepared samples revealed good electron‐transparent specimen quality, indicating the feasibility for using Xe^+^pFIB to prepare Al TEM samples. However, it was noted that curtaining and fast erosion of the sample surface including the Pt protective layer occurred at currents higher than 180 nA (30 kV) for rough milling and 1.8 nA (30 kV) for final milling. This rough milling current is significantly higher than typical rough milling currents usable in conventional Ga+FIB. The higher milling current of Xe+pFIB usable for TEM sample rough milling can be attributed to the better high‐current ion beam profile of Xe+pFIB comparing to Ga+FIB.^20^ No cavities or FIB‐induced precipitates were observed, even at the highest ion currents used up to 1.8 nA for final thinning in this study, indicating the energy of atomic collisions gained from the Xe^+^ beam was not sufficiently high to implant significant Xe into the Al. This could motivate the use of Xe^+^pFIB in other applications such as MEMS (microelectromechanical systems) patterning, nano‐fabrication, circuit editing etc.

#### 3.1.1 Ion irradiation‐induced damage

**FIGURE 2:**
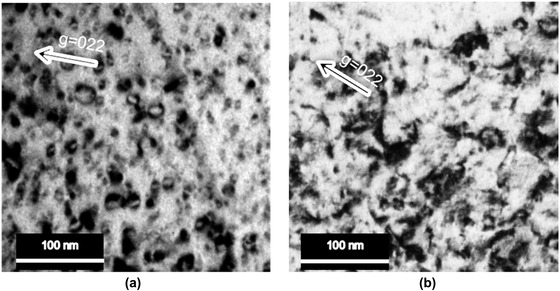
Bright‐field (BF) TEM images of (a) Ga^+^FIB and (b) Xe^+^pFIB prepared Al samples showing the high proportion of ion irradiation‐induced dislocation loops

TEM analysis of the defects generated by Ga^+^ and Xe^+^ ions in a [011]‐oriented grain of the pure Al sample (Figure 2) revealed the presence of dislocation loops and ‘black spot’ damage. No significant differences between Ga^+^FIB and Xe^+^pFIB generated defect microstructures were observed.

It is well known that vacancies and interstitials are created by ion bombardment of metals.^35^ According to SRIM (Stopping and Range of Ions in Matter) simulation using 10,000 ions, the energy Al received from Xe^+^pFIB at 30 kV is 4.79 keV/ion while from Ga+FIB it is 4.48 keV/ion.^36^, ^37^ SRIM simulations indicate that the ion cascades in Al by Xe^+^ ions are not as spread out as those produced by Ga^+^ ions due to the shorter range of Xe^+^‐induced recoils (Figure 4). In addition, the fraction of vacancies in the target Al that escape recombination or form immobile clusters may affect the dislocations generated in the materials.^38^ However, any local heating by Xe^+^ and Ga^+^ ion irradiation in Al could also play a role in the annealing of defects.^10^, ^39^ Thus, the defects observed in the final TEM specimen might be due to Ga and Xe at interstitial or substitutional sites whilst the amount of Ga and Xe implanted in the Al might be different. These combined factors may contribute to the final defects generated by Ga^+^FIB and Xe^+^pFIB.

#### 3.1.2 Amorphisation by Ga^+^FIB and Xe^+^pFIB

**FIGURE 3:**
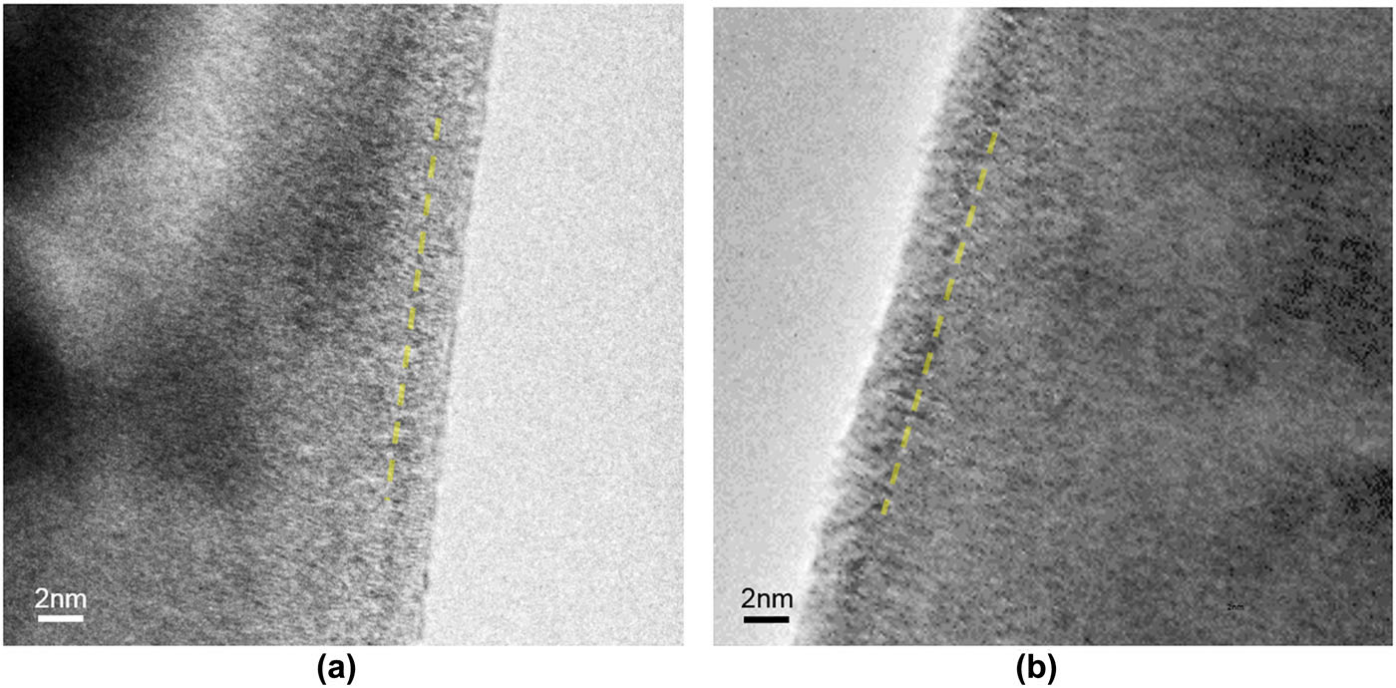
BF TEM images showing amorphisation induced by 0.23 nA 30 kV by milling: (a) Xe^+^pFIB, (b) Ga^+^FIB

**FIGURE 4:**
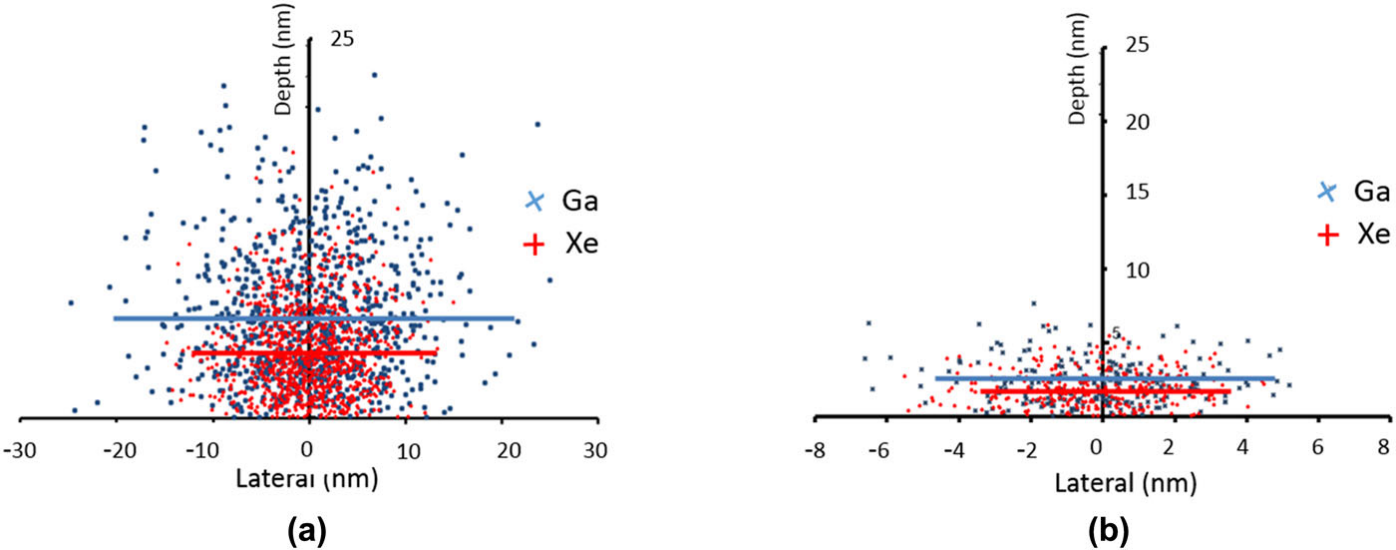
Simulated dimensions of Ga^+^ and Xe^+^ ion cascades in Al for implantation at (a) 30 kV and 89° incidence and (b) 5 kV and 89° incidence. The solid lines indicate the mean ion cascade depth and extent of lateral spread, and show that Ga^+^ ion cascades are deeper and wider than Xe^+^ ion cascades

Figure 3 shows images of the milled pin comparing the side wall amorphous layers induced by Ga^+^FIB and Xe^+^pFIB, both milled at 0.23 nA, 30 kV. SRIM calculations of the ion‐solid interaction at 30 kV with an 89° incident angle (Figure 4) and earlier work^5^ predicts that the amorphous layer produced by the Ga^+^ ion beam would be thicker than that produced by the Xe^+^ ion beam. However, the TEM evaluation of these specimens revealed very thin amorphous layers (∼ 3 nm) on both specimens, with an indication of slightly thinner layer for the Xe+ sample, although further HRSTEM analyses are required for quantitative confirmation. Amorphised layers induced by ion beam milling have been reported in several studies using both TEM and EBSD (electron backscatter diffraction).^24^, ^40^, ^41^ For example, Presley et al reported an amorphised layer with a similar 4 nm thickness for a 30 kV Ga^+^FIB milling of Al7075.^29^ Kelley et al^42^ compared amorphisation for Si between Ga^+^ and Xe^+^ and found Xe^+^ ions created a thinner amorphous layer, consistent with our results although the layers were much larger (21.5 nm with Ga^+^ ^42^) due to the nature of covalent bonds of Si and faster migration of ion‐induced lattice defects in Al.^43^

Typically, reducing the ion beam voltage can mitigate high‐energy ion beam‐induced defects,^44^, ^45^, ^46^ as shown in Figure 4, which shows that the ion cascade depth for 5 kV is shallower than that for 30 kV. Therefore, it is possible that the thickness of the amorphous layer and defects observed shown in Figure 2 may be reduced/eliminated if the final ion beam voltage is reduced. In addition, low‐voltage and low‐angle broad ion beam (BIB) technique can also be utilised to reduce the amorphous layer thickness.^34^, ^47^

#### 3.1.3 Compositional artefacts and redeposition

**FIGURE 5:**
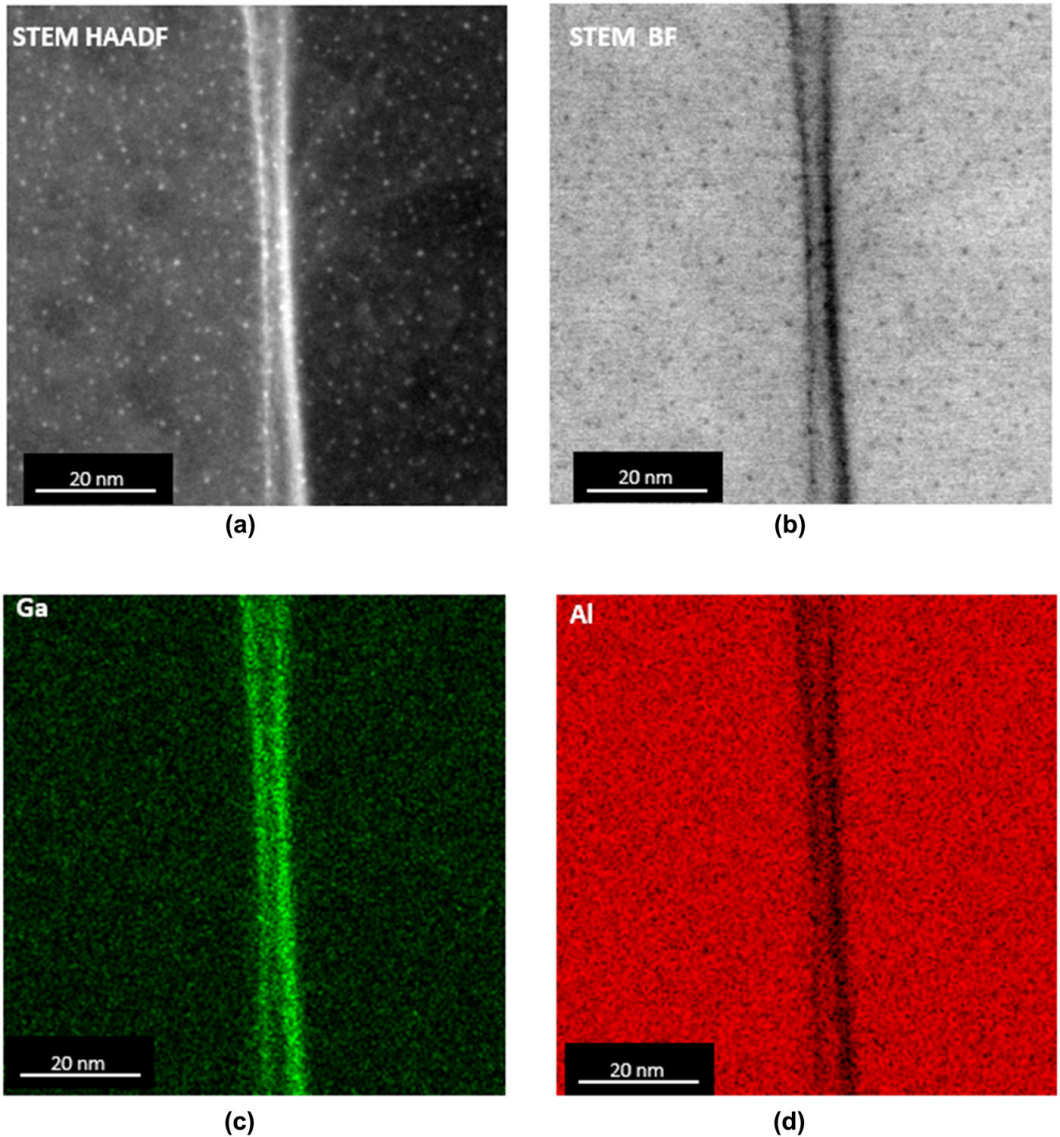
Slightly inclined grain boundary observed in a conventional Ga^+^FIB‐produced Al TEM sample: (a) HAADF STEM image showing two brightly imaging ‘lines’ and ‘dots’ within the grains caused by Ga segregation to the grain boundary and surface redeposition, respectively; (b) BF STEM image showing dark lines and dots caused by Ga segregation and surface redeposition, respectively; (c), (d) elemental maps for Ga and Al, respectively, extracted from the STEM‐EDX spectrum image dataset

The HAADF STEM image in Figure 5 shows a grain boundary (GB) decorated with Ga in the Ga^+^FIB generated sample. Ga was detected at most of the GBs in the polycrystalline Al sample generated by the conventional Ga^+^FIB TEM sample preparation procedure. The apparent ‘double line’ in Figures 5(a) and (b) delineates the intersection of an inclined grain boundary with the foil surfaces and the enrichment of Ga compared to Al, and indicates that the Ga concentration was particularly high where the GB intersects the surface of the TEM sample.

As a consequence of ion‐solid collision, the Al atoms absorb energy from energetic ions, local regions of lattice become highly distorted and contain high concentrations of vacancies form in the collision cascade.^37^ GB decoration of Ga is the result of the vacancy‐impurity binding energy and the large negative vacancy binding energy of the GB sites. Rajagopalan et al^48^ have shown that there is a very strong correlation between vacancy binding and Ga segregation for nearly all boundaries in Al. Due to the binding tendency of Ga in Al and the number of vacancies created when milling at grazing angles where collision cascades are localised near the top surface, it is not surprising to see that Ga is concentrated predominantly at top surface.

The vacancy‐impurity binding energy B in Al can be determined from the following equation^49^

(1) $$\frac{C}{\left(1-C\right)}=\;Ae^{\left\{\frac{-2B\left(1-2C\right)}{\mathrm{kT}}\right\}},$$

where C is the solid solubility of the impurity, *k* is the Boltzmann constant and *T* is the absolute temperature. The constants *A* related to vibrational entropy is 2.7.^49^ Taken the solubility of Ga in Al is 9 at.% according to the Al‐Ga phase diagram,^26^ the relationship of vacancy‐impurity binding energy to temperature can be simplified as *B* = 1.11 × 10^−23^ T. Thus, lowering the temperature of the specimen is another way of reducing Ga contamination. In this context, we anticipate cryo‐FIB might be an alternative route to reduce Ga^+^FIB‐induced compositional artefacts in Al.^9^, ^50^, ^51^, ^52^

The speckled appearance of the specimen shown in Figure 5A is caused by Ga^+^FIB redeposition leading to Ga‐containing nanoparticles on the TEM FIB lamella surfaces.^53^ These bright spots in the HAADF STEM image (Figure 5a) might be due to Z‐contrast as Ga has a higher atomic number compared to Al or mass thickness contrast caused by the local increase in sample thickness caused by the redeposition. A higher vacuum environment in the FIB column may reduce redeposition by pumping away the debris in the atmosphere, but it is usually impossible to completely eradicate the problem. Nonetheless, a high vacuum in the FIB system, particularly in the final milling steps, is recommended when producing TEM specimens.

**FIGURE 6:**
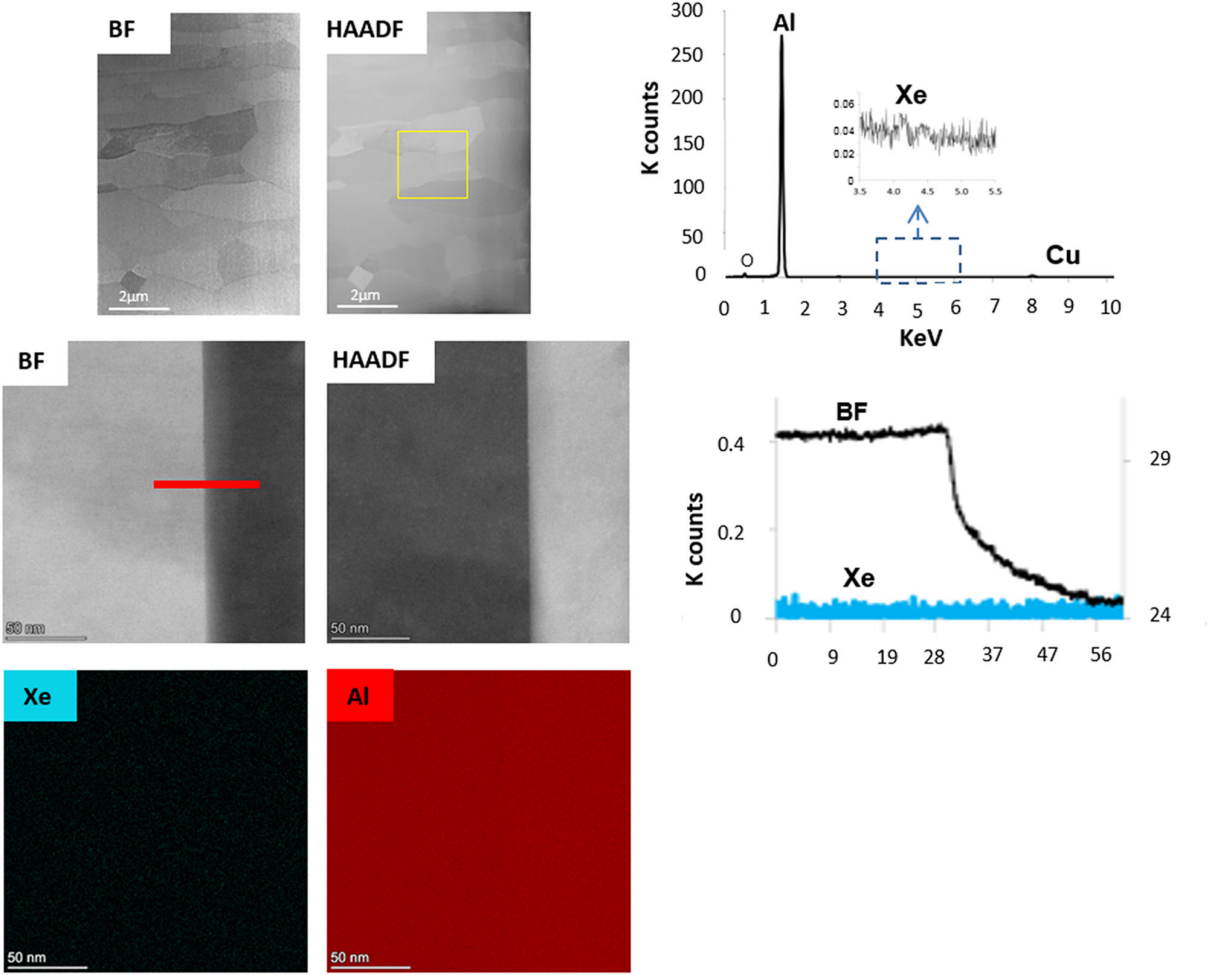
Xe^+^pFIB‐produced Al TEM sample. (a) STEM BF image; (b) complementary STEM HAADF image of the fine‐grained Al alloy; (c) STEM‐EDX sum spectrum collected over the area shown in (b) in the yellow box; (d) BF STEM image; (e) complementary HAADF STEM image of a GB; (f) EDX line scan (yellow line) showing no Xe enrichment at the GB in (d); (g) STEM‐EDX maps of Al; (h) STEM‐EDX maps of Xe showing no Al depletion or Xe enrichments at the GB shown in (d)

In contrast to Ga^+^FIB‐prepared samples, the HAADF STEM image of the specimen prepared using the Xe^+^pFIB shows that the grain boundaries appeared ‘clean’ (Figures 6a‐d) compared to the Ga^+^FIB samples (Figure 5). STEM‐EDX line‐scans across the grain boundary and elemental maps obtained from the STEM‐EDX SI dataset show no change in composition (Figures 6a, e, f). The sum spectrum (Figure 6c) shows no significant Xe in the specimen, although a very small trace was detected. The low solubility of Xe in Al is consistent with the nearly undetectable concentration of Xe in the sample as measured by STEM‐EDX microanalysis. However, it is noted that Xe ion implantation may be possible at higher ion energies or with incident angles normal to the surface as observed by S Donnelly etc.^32^

pFIBIt was observed that Xe^+^pFIB‐produced samples generally have less redisposition compared to Ga^+^FIB generated samples; the BF TEM images in Figures 6(c)‐(d) of the Xe^+^ pFIB‐prepared specimen showed a lack of speckle contrast that was characteristic of the Ga FIB samples. To understand this difference, assuming the Xe^+^ and Ga^+^ ions carries the same voltage and charge (dosage), kinetic energy of the ions can be written as

$$\;\frac{{P_{Xe}}^{2}}{2m_{Xe}}=\frac{{P_{Ga}}^{2}}{2m_{Ga}}\;,$$

where *m* is the mass of the ion and *P* is the momentum. It can be simply derived as *P_Xe_* = 1.37 P_Ga_. Assuming the Xe ion and Al atom have a rigid body elastic collision, the momentum transferred to an Al atom with downward vector and large magnitude of momentum helps ‘push away’ the sputtered atoms giving them less chance to redeposit onto the freshly milled surface. On the other hand, inherent material properties like adhesion, which is the degree of absorption of the redeposited atoms onto the milled surface, might play an important role. We cannot exclude a chemical reaction of Ga with Al during milling in the Ga^+^FIB. This might contribute to the particulate reaction products (redeposition materials) with greater affinity to the Al lamella as well. All these hypotheses will be addressed in a further study using an Atomic force microscope (AFM) and an aberration‐corrected STEM characterisation studies.

### 3.2 AA7108‐T6 alloy TEM samples

**FIGURE 7:**
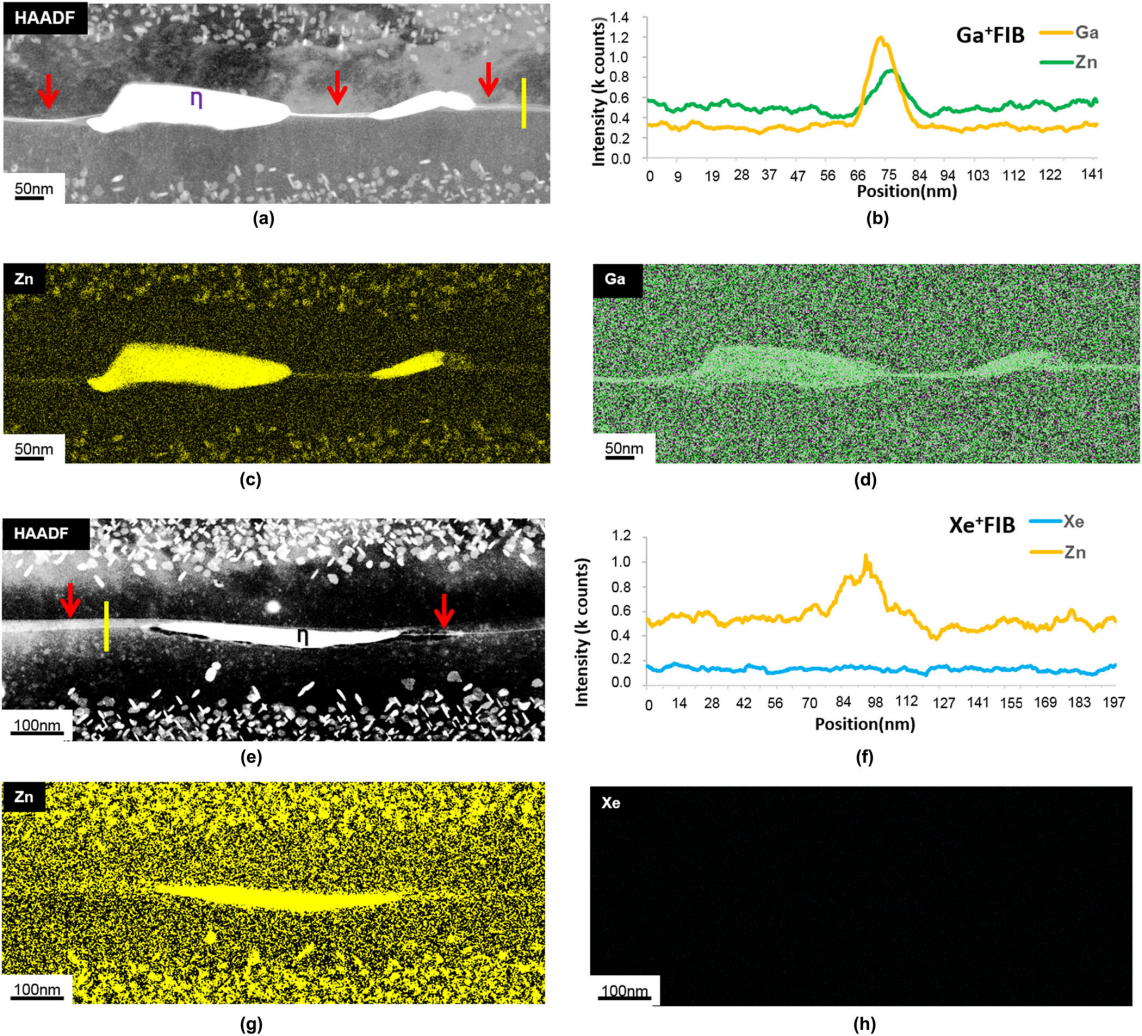
STEM‐EDX analysis of grain boundaries in (a)‐(d) for Ga^+^FIB and (e)‐(h) for Xe^+^pFIB‐produced AA7108‐T6 specimens. (a) HAADF STEM image of a GB region; (b) STEM‐EDX line scan across the GB (yellow square) in (a) showing the presence of Ga; (c) and (d) STEM‐EDX maps of Zn and Ga, respectively; (e) HAADF STEM image of a GB region; (f) STEM‐EDX line scan across the GB (yellow square) in (e) that showed no detectable Xe precipitation; (g) and (h) STEM‐EDX maps of Zn and Xe, respectively

Figure 7 is the comparison results of the TEM/STEM data obtained from grain boundaries in the Al‐Mg‐Zn samples prepared by conventional Ga^+^FIB and Xe+pFIB. The HAADF STEM image for the conventional Ga^+^FIB sample exhibited bright contrast associated with the boundary (red arrow). This bright contrast persisted with increasing stage tilt indicating that it was caused by mass thickness contrast. However, it is unclear if the contrast at the GB in Figure 7(a) was due to Ga or to Zn‐rich intergranular precipitates MgZn_2_. STEM‐EDX microanalysis revealed that both Ga and Zn were present at the grain boundary (Figures 7a‐b). However, in the Xe^+^pFIB‐produced sample, there was no detectable Xe segregation at or near the grain boundary (Figure 7d), but Zn was unequivocally detected decorating the GB (Figure 7c). Clearly, the use of the Xe^+^pFIB eliminates the Ga implantation artefact, thereby minimising misleading data interpretation. Nevertheless, we did observe that some of our samples had very small traces of Xe, which might be due to variation of ion incidence angle during final polishing; hence, care is needed during milling process, and FIB ion implantation concentrations at or below the STEM‐EDX detection sensitivity cannot be ruled out. Encouragingly, these very small traces of Xe detected in some of our samples were not segregated at grain or phase boundaries.

**FIGURE 8:**
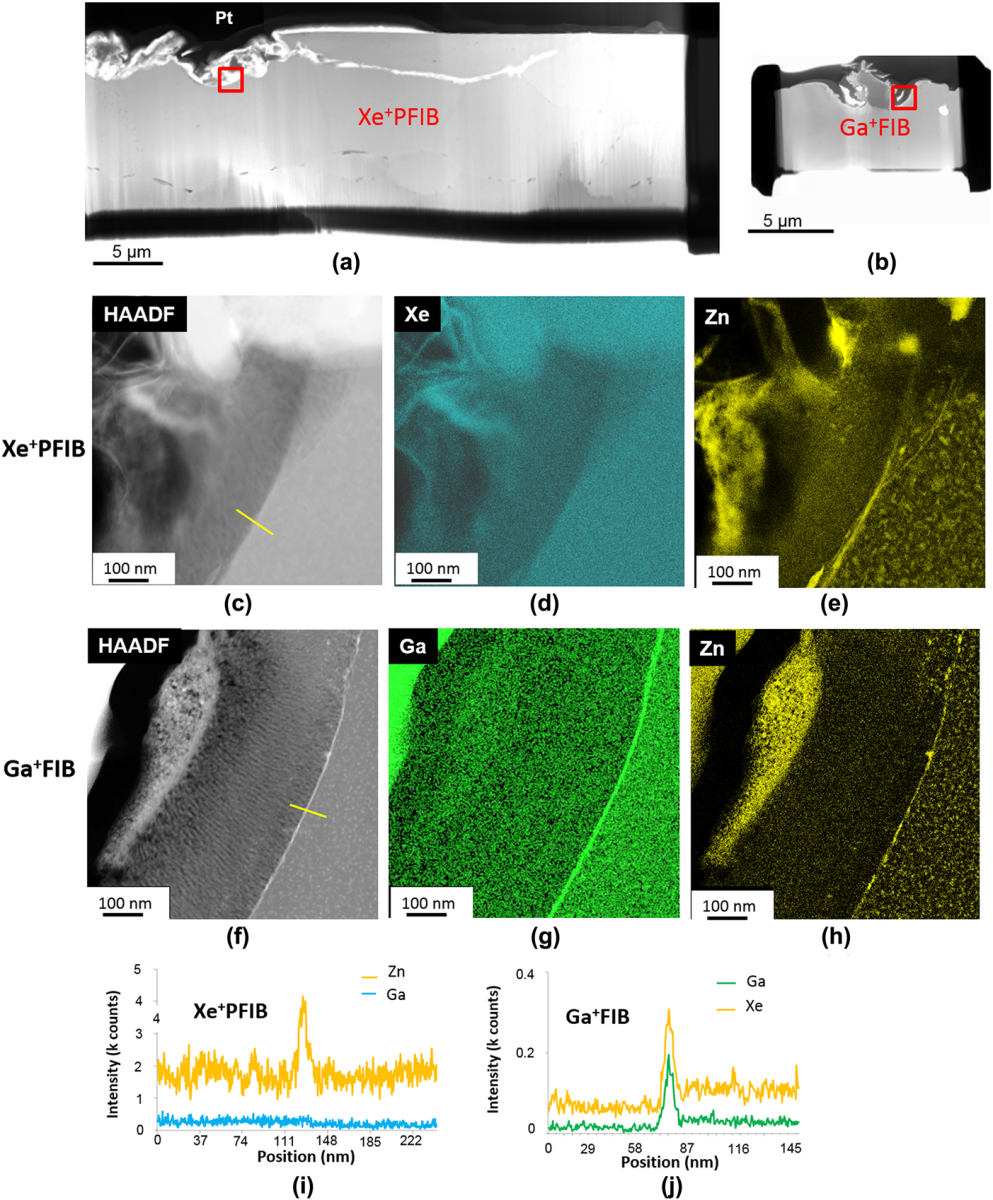
FIB specimens prepared from the as‐corroded AA7108‐T6 alloy. (a) STEM‐BF image of the Xe^+^pFIB‐prepared sample. (b) STEM‐BF image of the Ga^+^FIB‐prepared sample. (c) HAADF STEM image of the corrosion front in (a). (d), (e) STEM‐EDX maps for Xe and Zn, respectively, showing no Xe enrichment at the corrosion front but the presence of a Zn‐enriched layer. (f) HAADF STEM image of the Ga^+^FIB‐prepared sample shown in (b). (g), (h) STEM‐EDX maps for Ga and Zn showing enrichments for both Ga and Zn at the corrosion front. (i) STEM‐EDX line scan (yellow line in C) for Xe^+^pFIB sample showing Ga and Zn enrichments at the corrosion front, and (j) STEM‐EDX line scan (yellow line in F) for Xe^+^pFIB sample showing the absence of Xe enrichment and the presence of a Zn‐enriched layer at the corrosion front. Note that the BF STEM images are presented at the same magnification to further emphasise the increased specimen size of the pFIB sample

Figure 8 presents the comparison results of Xe^+^pFIB‐ and Ga^+^FIB‐prepared TEM specimens for microstructural analysis of the trenching corrosion site in AA7108‐T6, with a suspected Zn enrichment layer at the corrosion front interface between Al matrix and corrosion product. Such a complex, fragile and tortuous local specimen geometry is an ideal challenge for comparing the capabilities of the two FIB instruments, and is also highly important for both industrial and academic Al alloy corrosion research. The STEM‐EDX elemental maps (Figures 8e‐e) and line scans (extracted from the SI dataset) across the corrosion front (yellow line in Figure 8c) on the Xe^+^pFIB‐produced TEM sample revealed that there was no Xe enrichment at the oxidation front interface (Figures 8d‐e, i). This result is similar to our results for GBs in pure Al and AA7108‐T6. The STEM‐EDX maps (Figures 8g‐h) and line scan (Figure 8f) across the corrosion front of Ga^+^FIB‐produced sample is presented in Figure 8(j), which clearly shows the Ga enrichment at the interface in both the map and line scan. Hence, it is clear that only the Xe^+^pFIB‐prepared sample provided direct evidence that the bright line at the corrosion front in HADDF image is the result of Zn enrichment. No Zn enrichment was detected in the grain prior to the corrosion test. During corrosion, a Zn‐enriched layer formed at the corrosion front due to lower Gibbs free energy for Zn oxidation compared to that for Al, and can affect the local electrochemistry during corrosion.^54^

Similar to the results for the Al sample, no Xe ‘bubbles’, cavities, precipitates or other microstructural changes were observed at the corrosion front or within the microstructure of the Xe^+^pFIB‐prepared AA7108‐T6 TEM samples. These results appear to be encouraging in comparison to the study by Allen et al^32^ that showed Xe precipitates formed by directly implanting Xe+ into Al at 35 kV with a high dose.

Having demonstrated that Xe+pFIB technique can produce good TEM samples free of GB segregation artefacts, it is appropriate to consider the more general benefits compared to Ga+FIB sample preparation, namely the ability to prepare larger electron‐transparent specimen areas via high‐speed milling. The 50 μm × 15 μm TEM sample shown in Figure 8(a) was generated in Xe^+^pFIB in approximately 8 hours. This is a similar length of time to that required to produce a 15 μm × 5 μm TEM sample via Ga+FIB shown in Figure 8(b). The Xe^+^pFIB rough trench milling was performed at 180 nA and 30 kV, followed by 59 nA and 30 kV. Fine trench milling was performed at 15 nA (30 kV). The heavier Xe ions and higher beam currents available in Xe^+^pFIB enable the use of considerably faster milling rates compared to those for the conventional Ga^+^FIB; thus, significantly larger electron‐transparent samples can be produced in a shorter time. After the conventional lift‐out procedure,^16^ prethinning was performed at 1.8 nA followed by final thinning at 0.23 nA (all 30 kV). The relatively lower current of Xe^+^pFIB was used at final thinning steps because ion beam profile is generally broader than Ga^+^FIB. For the entire sample preparation process, final thinning steps took majority of the time.^55^ In comparison to a typical 200‐300 nm lamella thickness considered suitable for starting final thinning in the Ga^+^FIB process, the Xe^+^pFIB final milling starting thickness was around 500 nm due to the broad beam tail at lower currents compared to that of the Ga^+^FIB at the same current.^23^ In general, the trenching and lift‐out process took about 30 minutes; the entire process to generate a good TEM specimen lasted from 2 to 10 hours depending on the size of the final thinning area. The capability of Xe+pFIB for large area, thick lift‐out also provides the opportunity for more complex experiments with Al alloys beyond TEM specimen preparation, including 3D imaging with SEM‐EDX or EBSD, and SIMS sample preparation.^18^, ^56^, ^57^

## 4 CONCLUSIONS

Various artefacts introduced in TEM samples of Al prepared by Ga^+^FIB and Xe^+^pFIB have been investigated. For both pure Al and the commercial Al‐Zn‐Mg alloy (AA7108‐T6) the Xe^+^pFIB route is superior compared to conventional Ga^+^FIB for Al TEM samples in the following respects:

1. Xe^+^ pFIB milling produced relatively ‘clean’ surfaces with no Xe enrichments detected at GBs or phase boundaries in any of the Al samples whereas Ga enrichments at GBs and in their vicinity were observed as a result of the conventional Ga^+^FIB milling process, as expected.
2. Xe^+^pFIB milling generated a similar level of ion‐induced damage compared to conventional Ga^+^FIB milling process.
3. Both Xe^+^pFIB and conventional Ga^+^FIB thinning resulted in very thin amorphous layer after final milling at 0.23 nA/30 kV, although the Xe+pFIB sample appeared to have a slightly thinner layer, which merits further study.
4. The surface nanoparticle artefacts resulting from redeposition induced by Ga^+^FIB were less present for the Xe^+^ pFIB sample.
5. The efficiency of preparing TEM lift‐out specimens is greatly improved using dual beam Xe^+^pFIB, enabling much substantially larger electron‐transparent areas than is possible with the conventional Ga^+^FIB. Higher current can be used for rough milling process; however, the overall speed of TEM sample preparation using the Xe^+^pFIB was found to be limited by the need to use low milling currents in the final thinning process.

We propose that further improvements could be realised by improving Xe^+^pFIB beam profile at low and high currents and by using cryo‐FIB to attempt to reduce preparation artefacts in the Al samples.
